# Sacrococcygeal teratoma in an adult female patient—case report and review of literature

**DOI:** 10.1093/jscr/rjad731

**Published:** 2024-01-18

**Authors:** Mohammad I Alsahouri, Qusai A Alsalah, Arein A Abufara, Ahmad G Hammouri, Ibrahim Alzatari, Usama Qumsieh

**Affiliations:** Faculty of Medicine, Palestine Polytechnic University, Hebron 9020000, Palestine; Faculty of Medicine, Palestine Polytechnic University, Hebron 9020000, Palestine; Faculty of Medicine, Palestine Polytechnic University, Hebron 9020000, Palestine; Radiology Department, Al-Ahli Hospital, Hebron 9020000, Palestine; Radiology Department, Al-Ahli Hospital, Hebron 9020000, Palestine; Faculty of Medicine, Palestine Polytechnic University, Hebron 9020000, Palestine; Pediatric Surgery Department, Al-Ahli Hospital, Hebron 9020000, Palestine

**Keywords:** sacrococcygeal, teratoma, adult, coccygectomy

## Abstract

Sacrococcygeal teratoma (SCT) in adults is very rare with only a few cases documented in the literature, adult prevalence varies between 1 in 40 000 and 1 in 63 000. Most SCTs are located either mainly extra-pelvic (types I and II), which are more commonly seen in neonates; however, mainly intra-pelvic tumors (types III and IV) are more typical in adulthood. Extra-pelvic teratomas are extremely rare in adults. When SCT manifests in an adult, it appears as a slow-growing tumor without symptoms and usually manifests after becoming large enough to cause compression symptoms. SCT has a 1–2% probability of malignant transformation. Herein, we report a 20-year-old female, who was diagnosed with lower back swelling since childhood that increased in size over the last 2 months; imaging revealed an extra-pelvic mass. This case describes an atypical scenario for SCT, which was successfully managed with surgery. The histopathology report confirmed the diagnosis.

## Introduction

Sacrococcygeal teratoma (SCT) is usually a benign tumor that develops at the base of the coccyx and is believed to arise from remnants of the primitive streak that contains layers of pluripotent embryonic germ cells from any of the three primitive cell layers [1]. There’s no known cause for teratomas yet [2]. Children frequently get these tumors, which can also be detected prenatally [3], but they are extremely uncommon in adulthood [4] with only a few cases documented in the literature [5]. Adult incidence ranges from 1 in 40 000 to 1 in 63 000, with a 3:1 female-to-male ratio [2, 6, 7]. According to their location, Altman divided SCTs into four types according to the anatomical configuration: extra-pelvic (types I and II) tumors that are more commonly seen in neonates, with a small number of mainly intra-pelvic tumors (types III and IV) that are more typical in adulthood [8]. Adult cases typically present as intrapelvic masses, in contrast to newborns, which typically occur as extra-pelvic SCTs in over 90% of cases [6]. Herein, we present a 20-year-old female who presented with lower back swelling because of type I SCT that was managed successfully with surgery. This report aims to shed light on this rare entity and alerts surgeons not to oversee SCT in atypical cases.

### Case presentation

A 20-year-old female was diagnosed with a gradually enlarging lower back swelling. The patient mentioned having this swelling since childhood, and experiencing mild discomfort while sitting. However, medical advice was not sought until adulthood when the swelling became more prominent and associated with pain and numbness radiating into both lower limbs two months ago. She had no abnormal discharge, nausea, vomiting, change in bowel habits or urinary symptoms.

Upon physical examination, a large, irreducible swelling was observed at the lower back, with no skin discoloration, wounds, or discharge. The neurological exam showed a power of 5/5 in both lower limbs. Additionally, serum tumor markers including beta-human chorionic gonadotropin and alpha-fetoprotein were normal.

**Figure 1:**
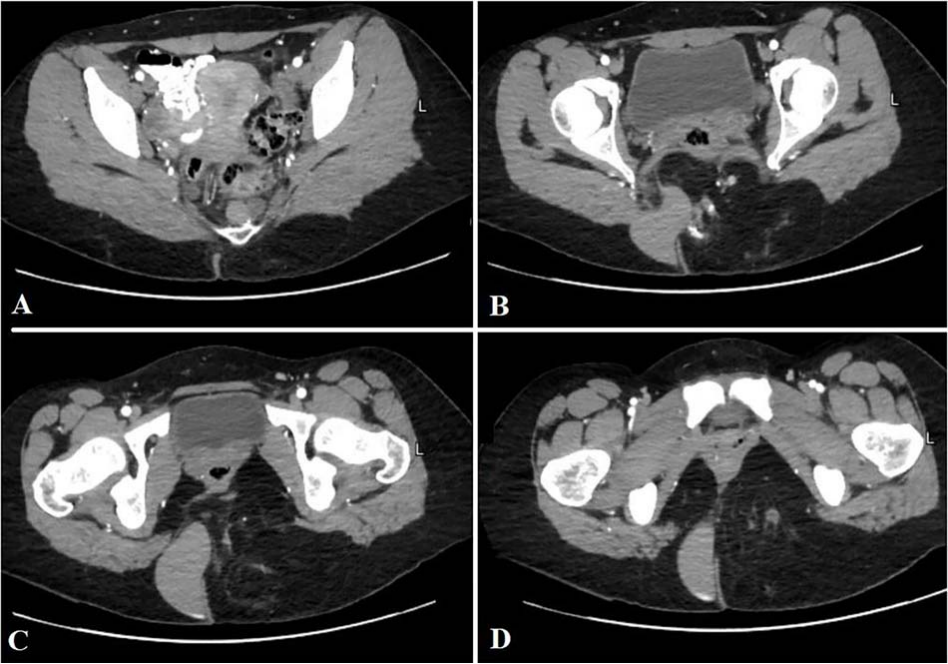
Selected axial CT cuts of the pelvis with oral and IV contrast (Arterial phase) from above downward (A–D), showing the mentioned gluteal mass measuring about 9 × 15 × 0.5 cm^3^. The boundaries of the lesion were not clearly defined and the dimensions were assumed based on its mass effect on the adjacent structures. The lesion consisted of mixed tissues (fat predominantly, fluid and calcifications). The fluid portion appeared on the right aspect of the gluteal region with high density, indicating mucinous/proteinaceous content. Extensions into the ischio-rectal fossa are noted with minimal pre-coccygeal components seen as well.

**Figure 2:**
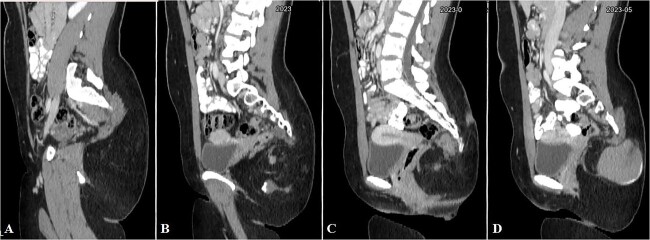
Selected sagittal CT cuts of the pelvis with oral and IV contrast (Venous phase) from left to right sides of the body (A–D), showing the mentioned gluteal mass. The cystic component of the mass is noted at the right gluteal region and the pre-coccygeal portion is well shown in C.

Contrast-enhanced computed tomography (CT) showed an ill-defined mass-like lesion in the gluteal region. The lesion consists of mixed tissue (fat predominantly, fluid and calcifications) and is seen exerting mass effect on the adjacent muscles and extending into the ischioanal, ischiorectal fossa and pre-coccygeal region (Figs 1 and 2).

**Figure 3:**
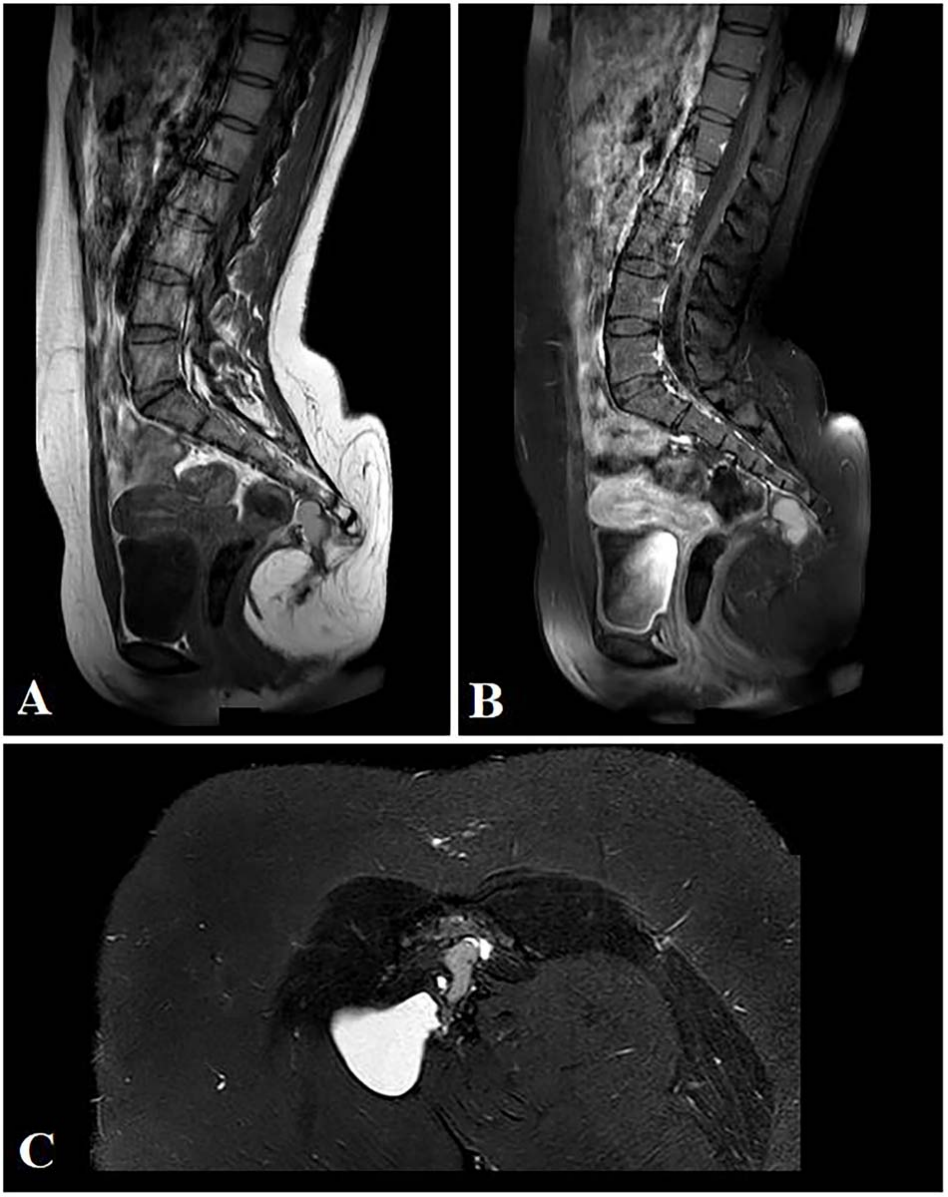
Selected sagittal MRI images (T1-sequence) without (A) and with contrast (B), both showing the extension of the mass with a visible pre-coccygeal component. The tumor is seen composed of mixed solid (predominantly fat) and cystic (fluid) components. The cystic component appears hyper-intense on the STIR sequence (C).

Magnetic resonance imaging (MRI) showed that the mass is mainly extra-pelvic and composed of mixed cystic and solid components. Minimal pre-coccygeal extension is noted suggesting type 1 SCT (Fig. 3).

The patient underwent a surgical excision of the mass through a sacral elliptical incision. The mass was excised along with the coccyx. The operative and postoperative courses were uneventful, and the patient was discharged two days after the surgery in good general condition. The excised mass was sent for pathological examination.

A gross examination of the cut surface of the mass showed multiple cysts containing variably turbid and mucoid fluid. Otherwise, the cut surface was fibrofatty tissue. Histopathology confirmed the diagnosis of a mature, benign teratoma with no evidence of malignancy.

## Discussion

SCTs are the most common type of fetal tumors, and they account for about half of all teratomas in children [4]. SCTs have an unclear etiology; however, they are hypothesized to arise from the migration of totipotent stem cells to the coccygeal region [2]. SCTs are divided into Types I–IV according to tumor location based on criteria proposed by Altman (Table 1) [8]. Altman type III is the most prevalent in adults [3, 9], whereas type I and II in newborns are the most common [10]. Histologically teratomas are divided into three categories: mature, immature, and malignant [2]. The majority of teratomas are benign, with about 12% having the potential to transform into malignant forms such as squamous cell carcinoma, adenocarcinoma, sarcoma, and other malignancies [11]. Altman type I in newborns and children have the lowest risk of malignancy, while type IV has the highest potential [12].

**Table 1:** Sacrococcygeal teratoma types based on tumor location (Altman’s criteria) [8]

Type	Percentage	Description
Type I	46%	Totally outside the pelvis
Type II	35%	Predominantly outside the pelvis but with a small number of intrapelvic components
Type III	9%	Mostly inside the pelvis but with a small number of external components
Type IV	10%	Completely inside the pelvis

Most patients with SCTs are asymptomatic or present with compression symptoms such as constipation, sacrococcygeal pain and bladder dysfunction [3]. Ultrasonography is an initial diagnostic technique for evaluating cystic and solid components; however, it is sometimes inconclusive in detecting intrapelvic extension [5]. CT and MRI scans are very useful in determining the area of the tumor and the degree of invasion, which assists in guiding the surgical approach [3]. Tumor markers may increase in malignant pathological tissue mainly within immature teratomas and malignant transformation of mature teratomas [13].

The suggested first-line therapy for SCTs is total surgical excision by a trans-abdominal, trans-sacral, or both trans-sacral and abdominal approaches, depending on the tumor’s anatomical position [9], The removal of the coccyx during surgery is controversial, as the primary justifications for coccyx resection are large tumors that may require exposure during surgery. In addition, SCTs emerge from pluripotent cells of the coccyx; tumors may adhere to the coccyx, and coccyx excision can prevent recurrence [2]. Surgical complications include excessive bleeding, bowel/urinary dysfunction and dysesthesia [12]. In 2011, it was the first time an adult patient was treated with laparoscopic surgery for SCT [9]. Additional treatment with chemotherapy and radiation is indicated in malignant patients. The conventional treatment regimen has not been thoroughly defined due to the entity’s rarity [10]. in our case, the patient was successfully managed with a trans-sacral approach with coccygectomy without any complications.

Risk factors for recurrence include immature components, frank malignancy and incomplete resection. The probability of tumor recurrence is reported to be 30–40% on surgery without concurrent coccyx removal [12]. Additionally, the overall recurrence rates for adult mature and immature SCT are 10 and 20%, respectively [10]. With complete surgical excision, benign SCT has a great prognosis; however, malignant SCT has a poor prognosis [10].

## Conclusion

Although SCT is exceptionally rare in adults, it should be considered in the differential diagnosis for patients presenting with unexplained lower back swelling. Clinicians may encounter challenges in diagnosing SCT due to the lack of specific signs and symptoms. Several factors may contribute to a delayed diagnosis, including the delay in seeking medical care, the lack of symptoms in most adult cases and the absence of serious complications.

## Data Availability

The data used to support the findings of this study are included in the article.
